# Raptinal bypasses BAX, BAK, and BOK for mitochondrial outer membrane permeabilization and intrinsic apoptosis

**DOI:** 10.1038/s41419-019-1790-z

**Published:** 2019-07-19

**Authors:** Sina Heimer, Gertrud Knoll, Klaus Schulze-Osthoff, Martin Ehrenschwender

**Affiliations:** 1 Department of Oral and Maxillofacial Surgery, University Hospital Regensburg, Franz-Josef-Strauss-Allee 11, 93053 Regensburg, Germany; 2 Institute of Clinical Microbiology and Hygiene, University Hospital Regensburg, Franz-Josef-Strauss-Allee 11, 93053 Regensburg, Germany; 3 Interfaculty Institute for Biochemistry, University of Tübingen, 72076 Tübingen, Germany; 4 German Cancer Research Center (DKFZ), 69120 Heidelberg, Germany

**Keywords:** Cancer, Apoptosis

## Abstract

Most antineoplastic chemotherapies eliminate cancer cells through activation of the mitochondria-controlled intrinsic apoptotic pathway. Therein, BAX, BAK, and/or BOK function as the essential pore-forming executioners of mitochondrial outer membrane permeabilization (MOMP). The activation threshold of BAX and BAK also correlates inversely with the required strength of an apoptotic stimulus to induce MOMP and thereby effectively determines a cell’s readiness to undergo apoptosis. Consequently, the ‘gatekeepers’ BAX and BAK emerged as therapeutic targets, but functional or genetic loss renders BAX/BAK-targeting strategies prone to fail. Here, we show that the small molecule Raptinal overcomes this limitation by triggering cytochrome c release in a BAX/BAK/BOK-independent manner. Raptinal exerts a dual cytotoxic effect on cancer cells by rapid activation of the intrinsic apoptotic pathway and simultaneous shutdown of mitochondrial function. Together with its efficacy to eliminate cancer cells in vivo, Raptinal could be useful in difficult-to-treat cancer entities harboring defects in the intrinsic apoptosis pathway.

## Introduction

Most antineoplastic chemotherapies rely on activation of the mitochondria-controlled intrinsic apoptotic pathway to eliminate cancer cells^1^. The key effector proteins for intrinsic apoptosis, BAX, BAK, and/or BOK, form (once activated) pores in the outer mitochondrial membrane and cause mitochondrial outer membrane permeabilization (MOMP)^2–4^. Subsequent cytochrome c release allows assembly of the ‘apoptosome’ complex^5^. This scaffold fosters activation of caspase-9, the prototypic initiator caspase of the intrinsic apoptotic pathway. Caspase-9 in turn activates the effector caspases 3 and 7, both executioners of apoptosis^6^. Notably, MOMP not only initiates the cascade-like activation of caspases. Concomitant loss of mitochondrial transmembrane potential also severely compromises the function of mitochondria. MOMP is therefore considered the point of no return and irrevocably condemns a cell to death. Not surprisingly, the ‘MOMP gatekeepers’ BAX/BAK and their interplay with the regulatory network of BCL-2 family proteins emerged as therapeutic targets in cancer therapy^4,7^. Direct pharmacological targeting of BAX/BAK or liberation from inhibitory BCL-2 family proteins ultimately aim to initiate intrinsic apoptosis^8–11^. For cytochrome c release and successful MOMP initiation, however, BAX/BAK-targeting strategies critically depend on functional pore-forming proteins and their readiness to be activated (also referred to as ‘mitochondrial priming’)^12^. Here, we report that Raptinal, a recently developed inducer of intrinsic apoptosis in vitro and in vivo^13^, overcomes this drawback. Raptinal rapidly triggers cytochrome c release in a BAX-, BAK-, and BOK-independent manner. Raptinal exerts a dual cytotoxic effect on cancer cells by rapid activation of the intrinsic apoptotic pathway and simultaneous shutdown of mitochondrial function. Difficult-to-treat cancer entities with defects in the intrinsic apoptosis pathway may thus still respond to Raptinal treatment.

## Results

### Raptinal rapidly triggers apoptosis in cancer cells

**Fig. 1:**
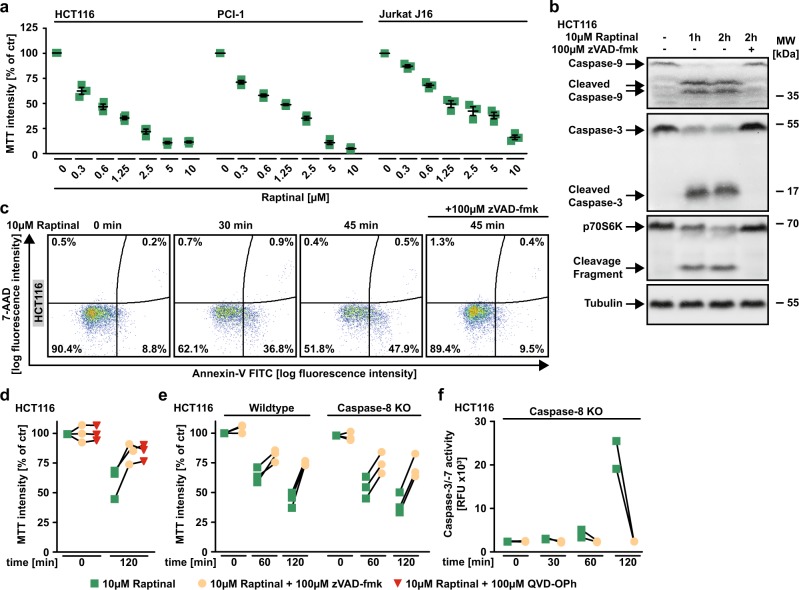
Raptinal triggers intrinsic apoptosis in various cancer cells. **a** HCT116, PCI-1 and Jurkat J16 cells were challenged with the indicated concentrations of Raptinal for 18 h. Data points and mean ± SEM from three independent experiments are shown. **b** HCT116 cells were challenged with Raptinal (10 µM) for the indicated periods of time in the absence and presence of the pan-caspase inhibitor zVAD-fmk (100 µM). After washing and lysis, western blot analyses were performed with antibodies specific for the indicated proteins. Detection of tubulin served as a loading control. **c** HCT116 cells were treated as in **b** and subsequently analyzed by flow cytometry for 7-AAD- and annexin-V positivity. For **b** and **c**, data shown are representative of two experiments performed. **d**, **e** HCT116 cells and caspase-8-deficient variants thereof were challenged with Raptinal (10 µM) for 60 min or 120 min in the presence and absence of the pan-caspase inhibitors zVAD-fmk (100 µM) or QVD-OPh (100 µM). **f** HCT116 Caspase-8 KO cells were treated with Raptinal (10 µM) for the indicated periods of time in the presence and absence of zVAD-fmk (100 µM). Caspase-3/-7 activity was assessed using the fluorogenic substrate (DEVD)_2_-R110. For **d**–**f**, individual data points of at least two independent experiments are shown. RFU, relative fluorescence units

Exposure to Raptinal showed cytotoxic effects in various cancer cell lines and triggered rapid processing of caspase-9 (Fig. 1a, b). Together with the observed cleavage of caspase-3 (a substrate of caspase-9) and p70S6K (a substrate of caspase-3), this indicated Raptinal-induced activation of the intrinsic apoptosis pathway^14^. Likewise, Raptinal-treated HCT116 cells stained positive for annexin-V and were rescued by the pan-caspase inhibitors zVAD-fmk and QVD-OPh (Fig. 1c, d). Deficiency of caspase-8, the initiator caspase of the extrinsic apoptotic pathway, had no protective effect and still allowed Raptinal-induced effector caspase activation (Fig. 1e, f). Taken together, our results are in agreement with the original description of Raptinal as a rapid inducer of apoptotic cell death via the intrinsic pathway^13^.

### BAX and BAK are dispensable for Raptinal-induced apoptosis

**Fig. 2:**
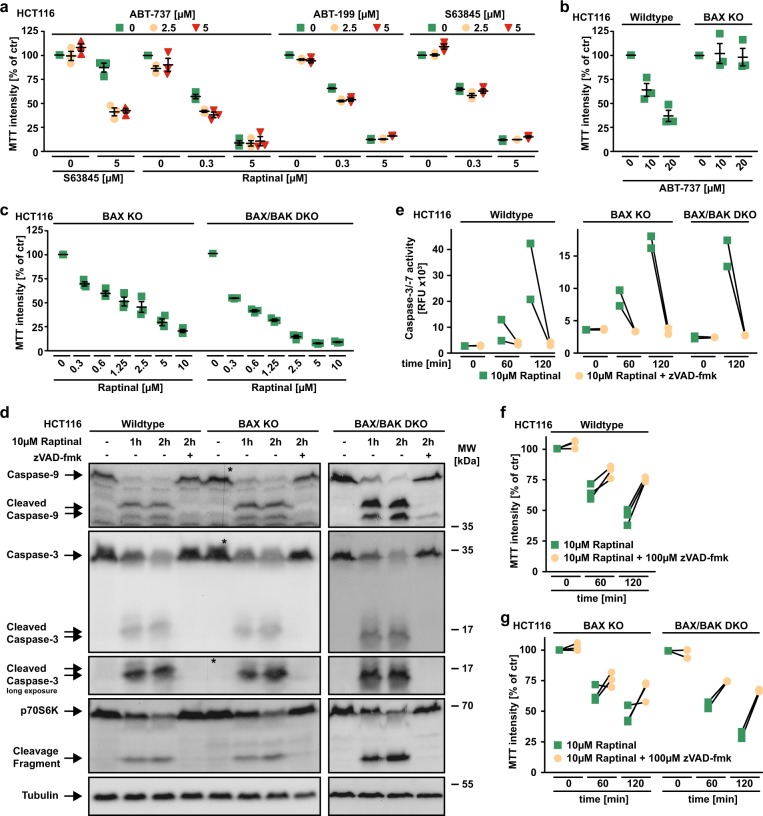
BAX and BAK are dispensable for Raptinal-induced caspase activation. **a** HCT116 cells were challenged for 18 h with the indicated concentrations of Raptinal and S63845 in the presence and absence of the indicated concentrations of ABT-199 (targeting BCL-2), ABT-737 (targeting BCL-2, BCL-XL and BCL-W), and S63845 (targeting MCL-1). **b**, **c** HCT116 cells and BAX- or BAX/BAK-deficient variants thereof were challenged with the indicated concentrations of ABT-737 and Raptinal for 18 h. **d** HCT116 cells and BAX- or BAX/BAK-deficient variants thereof were challenged with Raptinal (10 µM) for the indicated periods of time in the absence and presence of the pan-caspase inhibitor zVAD-fmk (100 µM). After washing and lysis, western blot analyses were performed with antibodies specific for the indicated proteins. Detection of tubulin served as a loading control. The asterisk (*) indicates a defect in the CCD sensor of the western blot imaging system. All samples were run on the same gel, no gels were sliced. **e** HCT116, HCT116 BAX KO and BAX/BAK DKO cells were treated with Raptinal (10 µM) for the indicated periods of time in the presence and absence of zVAD-fmk (100 µM). Caspase-3/-7 activity was assessed using the fluorogenic substrate (DEVD)_2_-R110. **f**, **g** HCT116, HCT116 BAX KO and BAX/BAK DKO cells were challenged with Raptinal (10 µM) for the indicated periods of time in the presence and absence of zVAD-fmk (100 µM). For **a**–**c** data points and mean ± SEM from three independent experiments are shown; for **d**, data shown are representative of two experiments performed; for **e**–**g**, individual data points of at least two independent experiments are shown. RFU, relative fluorescence units

Inhibition of antiapoptotic BCL-2 family proteins facilitates activation of the pore-forming proteins BAX and/or BAK and thus primes for intrinsic apoptosis^15^. The BH3 mimetic ABT-737 (targeting BCL-2, BCL-XL, and BCL-W) expectedly primed HCT116 cells for death induced by the MCL-1 inhibitor S63845. Surprisingly, neither S63845 nor ABT-199 (targeting BCL-2) nor ABT-737 acted synergistically with Raptinal in killing HCT116 cells (Fig. 2a).

**Fig. 3:**
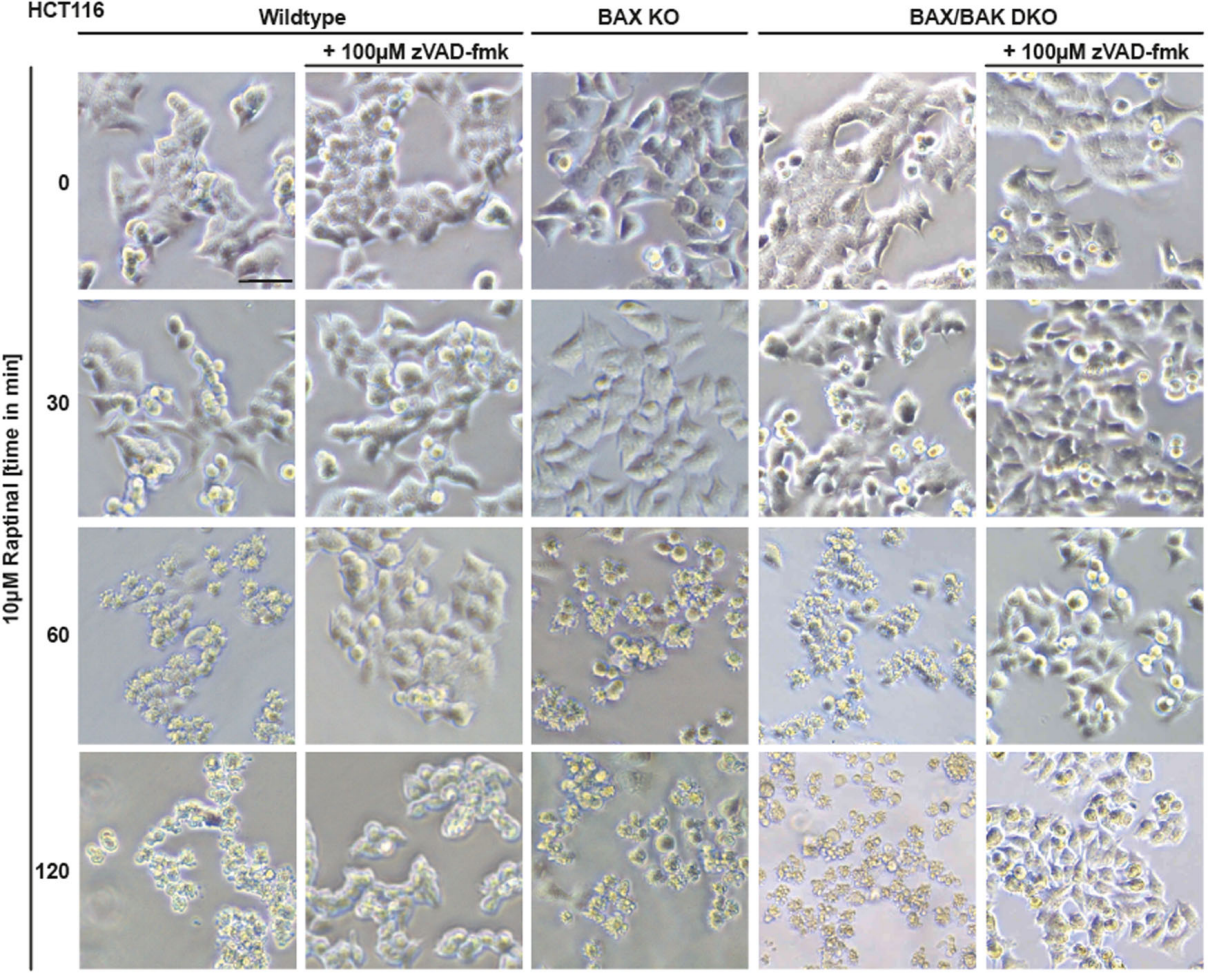
BAX/BAK-deficient cells display morphological signs of apoptosis upon Raptinal treatment. HCT116, HCT116 BAX KO, and BAX/BAK DKO cells were treated with Raptinal (10 µM) for the indicated periods of time in the presence and absence of zVAD-fmk (100 µM). Morphological changes were documented by bright field microscopy. Scale bar: 50 µm. Data shown are representative of two experiments performed

**Fig. 4:**
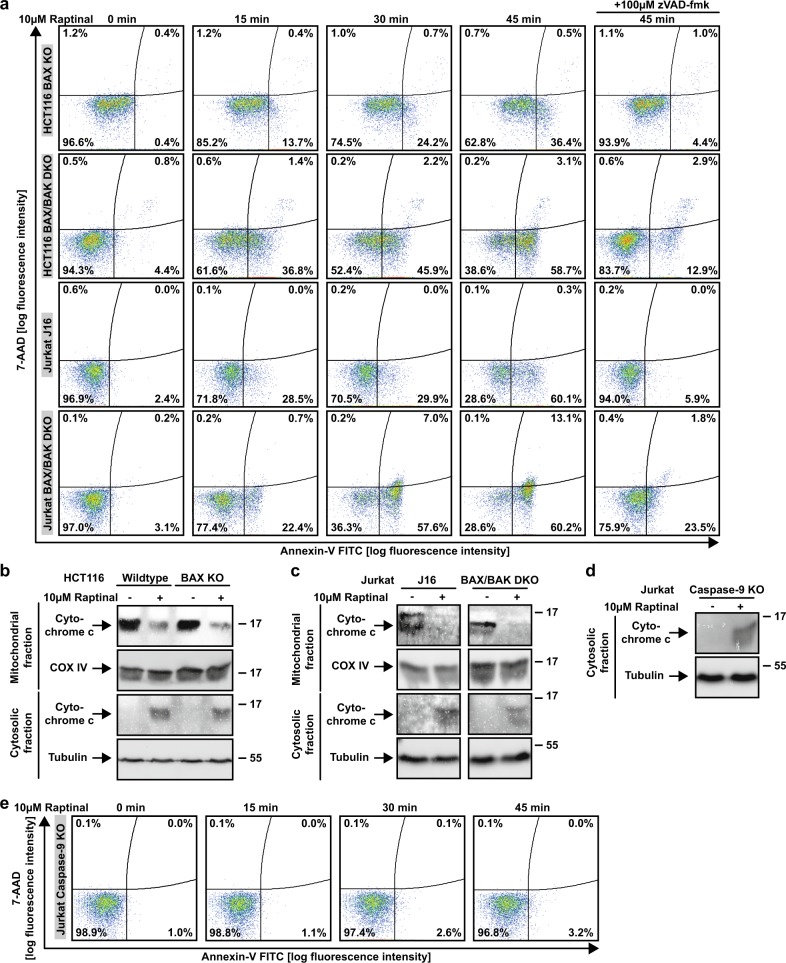
Raptinal causes cytochrome c release in the absence of the pore-forming proteins BAX/BAK. **a** HCT116 BAX KO, HCT116 BAX/BAK DKO, Jurkat J16 and Jurkat BAX/BAK DKO cells were challenged with Raptinal (10 µM) for the indicated periods of time in the presence and absence of the pan-caspase-inhibitor zVAD-fmk (100 µM). 7-AAD- and annexin-V positivity was analyzed by flow cytometry. **b–d** HCT116, Jurkat J16, Jurkat BAX/BAK DKO and Jurkat caspase-9 KO cells were challenged with Raptinal (10 µM) for 15 min (HCT116) or 30 min (Jurkat cells). After washing and lysis, western blot analyses were performed with whole cell lysates and mitochondria-containing fractions using antibodies specific for the indicated proteins. Detection of tubulin (whole cell lysate) and COX IV (mitochondria-containing fraction) served as loading control. **e** Caspase-9-deficient Jurkat cells were challenged with Raptinal (10 µM) for the indicated periods of time. 7-AAD- and annexin-V positivity was analyzed by flow cytometry. For **a**–**e**, data shown are representative of at least two experiments performed

The loss of BAX was sufficient to abrogate cytotoxicity of ABT-737 in HCT116 cells, whereas cytotoxicity of Raptinal was not depending on BAX or BAK (Fig. 2b, c). Proteolytic processing and activation of initiator and effector caspases was intact in Raptinal-treated BAX- and/or BAX/BAK-deficient HCT116 cells (Fig. 2d, e). Moreover, Raptinal rapidly caused ‘membrane blebbing’, a morphological hallmark of apoptotic cell death, irrespective of BAX/BAK (Fig. 3). Inhibition of caspase activity using zVAD-fmk expectedly abrogated Raptinal-induced apoptotic morphology (Fig. 3), reduced cytotoxicity (Fig. 2f, g) together with annexin-V/7-AAD-positivity in BAX/BAK-deficient and -proficient cells (Fig. 4a). Collectively, these results demonstrate that Raptinal triggers apoptotic cell death in the absence of the pore-forming proteins BAX/BAK.

### Caspase-9 propagates Raptinal-induced apoptosis after BAX/BAK-independent cytochrome c release

**Fig. 5:**
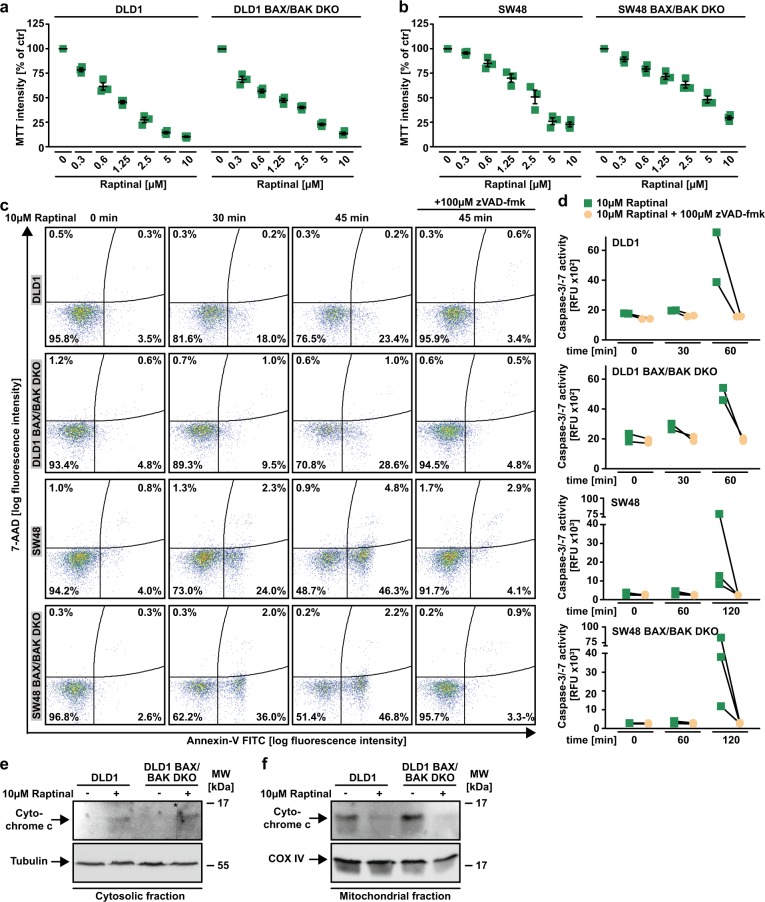
Raptinal induces intrinsic apoptosis in a variety of BAX/BAK-deficient cell lines. **a**, **b** DLD1 and SW48 cells and BAX/BAK-deficient variants thereof were challenged with the indicated concentrations of Raptinal for 18 h. **c** Cells were treated with Raptinal (10 µM) for the indicated periods of time in the presence and absence of the pan-caspase-inhibitor zVAD-fmk (100 µM). 7-AAD- and annexin-V positivity was analyzed by flow cytometry. **d** DLD1 and SW48 cells and BAX/BAK-deficient variants thereof were challenged with Raptinal (10 µM) in the presence and absence of the pan-caspase inhibitor zVAD-fmk (100 µM). Caspase-3/-7 activity was assessed using the fluorogenic substrate (DEVD)_2_-R110. **e**, **f** Cells were challenged with Raptinal (10 µM) for 60 min. After washing and lysis, western blot analyses were performed with whole cell lysates and mitochondria-containing fractions using antibodies specific for the indicated proteins. Detection of tubulin (whole cell lysate) and COX IV (mitochondria-containing fraction) served as loading control. The asterisk (*) indicates a defect in the CCD sensor of the western blot imaging system. All samples were run on the same gel, no gels were sliced. For **a** and **b**, data points and mean ± SEM from three independent experiments are shown. For **c**, **e**, and **f**, data shown are representative of at least two experiments performed. **d** shows individual data points of at least two independent experiments. RFU, relative fluorescence units

The loss of BAX/BAK is known to severely impair activation of the mitochondria-controlled apoptotic cascade^16,17^. The efficient Raptinal-induced caspase activation in BAX/BAK-deficient cells (Fig. 2d, e) could therefore either question an exclusive dependency of Raptinal on intrinsic apoptosis or point to an alternative, BAX/BAK-independent mechanism to initiate this pathway. Indeed, the latter seems the case as even in the absence of BAX/BAK Raptinal treatment resulted in cytochrome c release from the mitochondria (Fig. 4b, c). The loss of caspase-9 did expectedly not affect Raptinal-induced cytochrome c release (Fig. 4d), but conferred almost full-blown protection to Jurkat cells challenged with Raptinal (Fig. 4e). Importantly, we confirmed Raptinal-induced apoptosis in BAX/BAK-deficient DLD1 and SW48 cells to exclude cell line-specific effects (Fig. 5). In sum, our data support a model of fast Raptinal-induced activation of intrinsic apoptosis through a BAX/BAK-independent mechanism of cytochrome c release and subsequent caspase-9-dependent propagation of the death signal.

### BOK is dispensable for Raptinal-induced MOMP

**Fig. 6:**
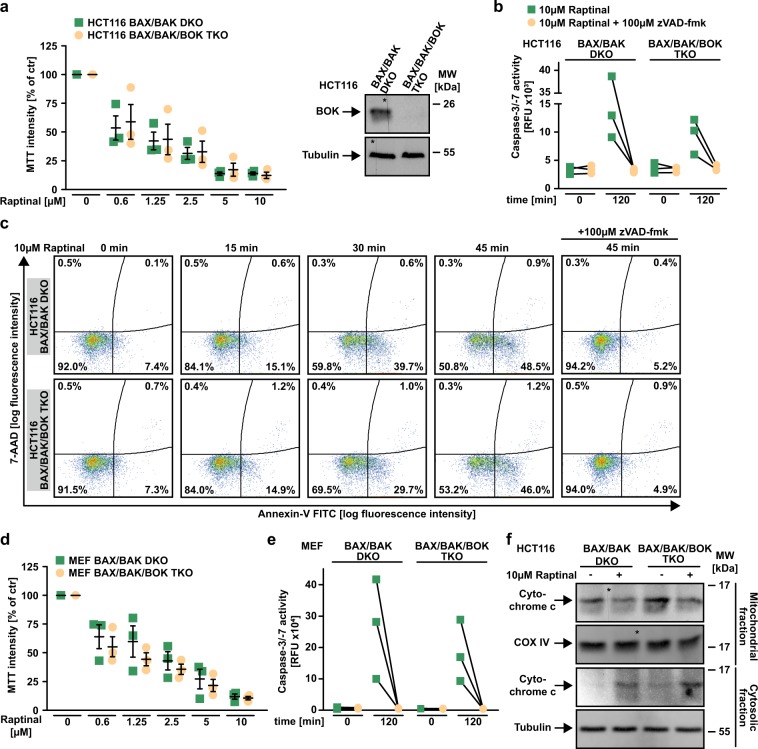
BOK is dispensable for Raptinal-induced MOMP. **a** Left panel: HCT116 BAX/BAK DKO and BAX/BAK/BOK TKO cells were challenged with the indicated concentrations of Raptinal for 18 h. Right panel: BOK levels were analyzed in lysates generated from HCT116 BAX/BAK DKO and BAX/BAK/BOK TKO cells by western blotting. The asterisk (*) indicates a defect in the CCD sensor of the western blot imaging system. All samples were run on the same gel, no gels were sliced. **b** HCT116 BAX/BAK DKO and BAX/BAK/BOK TKO cells were challenged with Raptinal (10 µM) for 120 min. Caspase-3/-7 activity was assessed using the fluorogenic substrate (DEVD)_2_-R110. **c** Cells were treated with Raptinal (10 µM) for the indicated periods of time in the presence and absence of the pan-caspase-inhibitor zVAD-fmk (100 µM). 7-AAD- and annexin-V positivity was analyzed by flow cytometry. **d** BAX/BAK- and BAX/BAK/BOK-deficient mouse embryonic fibroblasts (MEFs) were challenged with the indicated concentrations of Raptinal for 18 h. **e** BAX/BAK- and BAX/BAK/BOK-deficient MEFs were challenged with Raptinal (10 µM) for 120 min. Caspase-3/-7 activity was assessed using the fluorogenic substrate (DEVD)_2_-R110. **f** HCT116 BAX/BAK DKO and BAX/BAK/BOK TKO cells were challenged with Raptinal (10 µM) for 30 min. After washing and lysis, western blot analyses were performed with whole cell lysates and mitochondria-containing fractions using antibodies specific for the indicated proteins. Detection of tubulin (whole cell lysate) and COX IV (mitochondria-containing fraction) served as loading control. For **a** and **d**, data points and mean ± SEM from three independent experiments are shown. **b**, **e** show individual data points of three independent experiments. For **c** and **f**, data shown are representative of at least two experiments performed

BOK is another protein capable to form pores in the outer mitochondrial membrane and has been reported to induce cytochrome c release in the absence of BAX/BAK^18,19^. To clarify whether BOK is involved in Raptinal-induced MOMP, we challenged HCT116 BAX/BAK/BOK triple knock-out (TKO) cells and BAX/BAK/BOK-deficient mouse embryonic fibroblasts with Raptinal. In the absence of BAX, BAK, and BOK, Raptinal still exerted cytotoxic effects (Fig. 6a, d), induced caspase-3 and -7 activation (Fig. 6b, e), caused annexin-V/7-AAD positivity (Fig. 6c) and triggered cytochrome c release from the mitochondria (Fig. 6f). Collectively, these data argue against a dependency on BOK for Raptinal-induced intrinsic apoptosis.

### Raptinal-induced loss of mitochondrial function exerts caspase-independent cytotoxic effects

**Fig 7:**
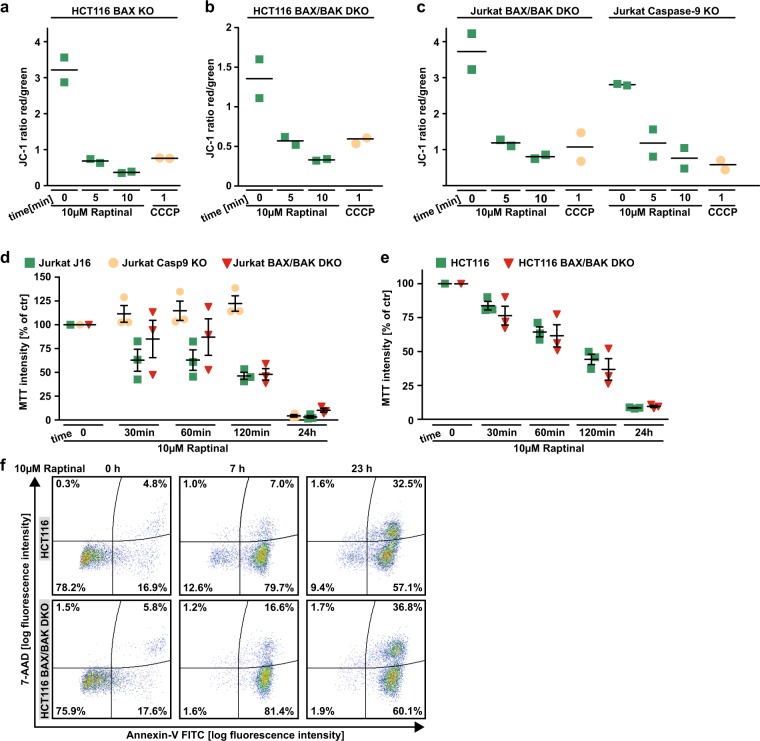
Raptinal impairs mitochondrial function. **a**–**c** HCT116 BAX KO, HCT116 BAX/BAK DKO, Jurkat BAX/BAK DKO, and Jurkat caspase-9 KO were treated with Raptinal (10 µM) for the indicated periods of time. Membrane potential of mitochondria was assessed by flow cytometry after staining with JC-1. CCCP treatment served as a positive control. Individual data points together with mean of at least two independent experiments are shown. **d** Jurkat J16, caspase-9 KO, and BAX/BAK DKO were challenged with Raptinal (10 µM) for the indicated periods of time. Cell viability was assessed by MTT staining. **e** HCT116 cells and BAX/BAK-deficient variants thereof were challenged with Raptinal (10 µM) for the indicated periods of time. Cell viability was assessed by MTT staining. For **d** and **e**, data points and mean ± SEM from three independent experiments are shown. **f** HCT116 and HCT116 BAX/BAK DKO cells were treated with Raptinal (10 µM) for the indicated periods of time. 7-AAD- and annexin-V positivity was analyzed by flow cytometry. Data shown are representative of two experiments performed

**Fig. 8:**
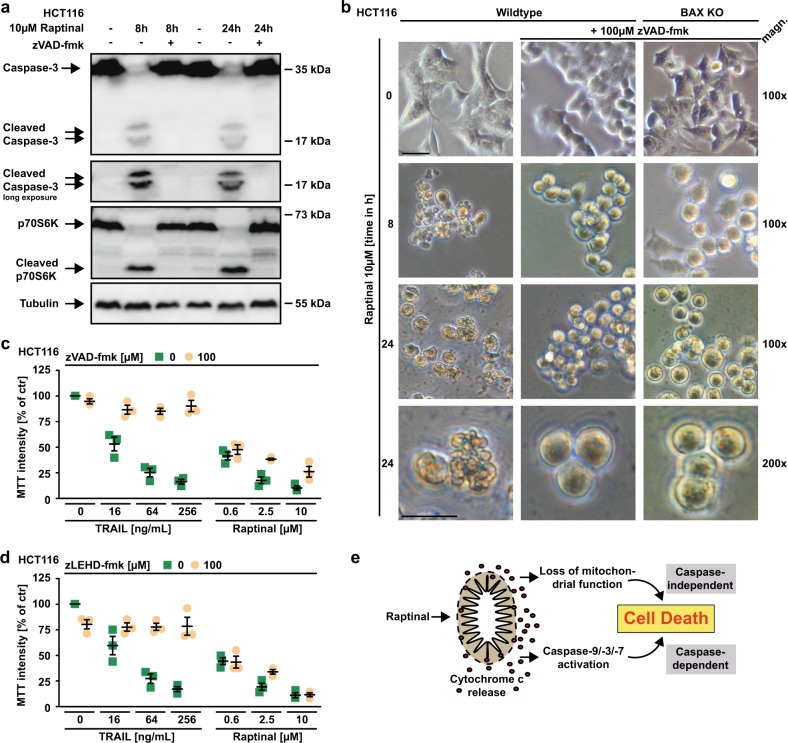
The loss of mitochondrial function complements caspase-dependent cytotoxicity of Raptinal. **a** HCT116 cells were challenged with Raptinal (10 µM) for the indicated periods of time in the absence and presence of the pan-caspase inhibitor zVAD-fmk (100 µM). After washing and lysis, western blot analyses were performed with antibodies specific for the indicated proteins. Detection of tubulin served as a loading control. **b** HCT116 and HCT116 BAX KO cells were challenged with Raptinal (10 µM) for the indicated periods of time in the presence and absence of zVAD-fmk (100 µM). Morphological changes were documented by bright field microscopy. Scale bar: 50 µm. HCT116 cells were challenged with the indicated concentrations of KillerTRAIL and Raptinal in the presence and absence of **c** the pan-caspase inhibitor zVAD-fmk (100 µM) or **d** the caspase-9-specific inhibitor zLEHD-fmk (100 µM) for 18 h. **e** Proposed model of Raptinal-induced cell death: Raptinal rapidly triggers cytochrome c release from the mitochondria in a BAX-, BAK- and BOK-independent manner. This determines a cell’s fate in two interdependent ways by (1) fast activation of caspases and subsequent apoptotic cell death or (2) the loss of mitochondrial function and caspase-independent cell death. For **a** and **b**, data shown are representative of two experiments performed. For **c** and **d**, data points and mean ± SEM from three independent experiments are shown

Apparently, Raptinal is capable to unleash the mitochondria-controlled death signal within minutes in a BAX/BAK/BOK-independent manner. Raptinal triggers release of cytochrome c from the mitochondria (Figs. 4b, c, 5f, and 6f) and thereby disrupts the electron transport chain. In line with rapid MOMP induction, 5 min of exposure to Raptinal was sufficient to decrease the mitochondrial membrane potential in BAX-, BAX/BAK- and caspase-9-deficient cells (Fig. 7a–c). Caspase-9-deficient cells showed almost full-blown protection when exposed to Raptinal for up to 2 h (Fig. 7d). Overnight treatment, however, was highly toxic in caspase-9- and BAX/BAK-deficient cells (Fig. 7d–f). Caspase inhibition efficiently blocked Raptinal-triggered caspase-3 activation even after 24 h (Fig. 8a), abrogated apoptotic morphology (such as membrane blebbing) of Raptinal-treated cells (Fig. 8b) and was expectedly sufficient to protect against TRAIL-induced extrinsic apoptosis (Fig. 8c). However, blocking caspase activity in HCT116 cells only partially relieved cytotoxicity of short-term (2 h) Raptinal treatment (Fig. 2f, g) and was even less protective upon long-term (24 h) exposure (Fig. 8c, d). Thus, MOMP induction and subsequent loss of mitochondrial function additionally exert caspase-independent cytotoxic effects.

Collectively, our data support a dual mode of action for Raptinal to determine a cell’s fate following BAX/BAK/BOK-independent MOMP (summarized in Fig. 8e): fast activation of the intrinsic apoptotic pathway (caspase-dependent cell death) and the loss of mitochondrial function (caspase-independent cell death).

## Discussion

Cancer cells differ widely in their threshold for activation of the intrinsic apoptotic pathway and consequently display striking differences in their susceptibility to chemotherapy-induced apoptosis. A new class of anti-cancer drugs called ‘BH3 mimetics’ disturbs the sophisticated network of BAX/BAK-regulating BCL-2 family proteins and increases the readiness for mitochondrial cytochrome c release. Essentially, BH3 mimetics prime mitochondria for death and reduce the minimally required strength of death-promoting stimuli to unleash mitochondrial apoptosis^10^. As ‘mitochondria-priming drugs’, BH3 mimetics show limited efficacy as single agents in most cancer entities, but act synergistically with conventional chemotherapies^15,20,21^. However, the absence of the pore-forming proteins BAX and/or BAK renders cancer cells refractory to both, mitochondrial priming and inducers of intrinsic apoptosis^10^. From the latter, Raptinal is a notable exception as (1) the mitochondrial priming of target cells does not enhance its cytotoxic activity (Fig. 2a) and (2) the cytochrome c release and MOMP occur in a BAX/BAK/BOK-independent manner (Figs. 4b, c, 5f, and 6f). Notably, Raptinal neither directly triggers cytochrome c release in isolated mitochondria nor via mitochondrial permeability transition pore (MPTP) formation in the inner mitochondrial membrane^13^. Earlier studies already noted that even in the absence of MPTP, BAX and/or BAK are not always required for mitochondrial cytochrome c release^22,23^. Our data also argue against a decisive role for BOK in Raptinal-induced MOMP (Fig. 6). Potentially, another yet unidentified mechanism for MOMP exists, which may involve specific lipids such as ceramide^24,25^.

Noteworthy, BAX/BAK/BOK-independent MOMP induction could also unlink mitochondrial priming from a cell’s readiness to activate the intrinsic apoptotic pathway. When MOMP is not executed through BAX/BAK-mediated pore-formation in the outer mitochondrial membrane, disturbing BAX/BAK-regulatory BCL-2 family protein interaction (e.g., using BH3 mimetics) may have no effect on the apoptotic threshold. Raptinal could therefore be effective in cancer cells with no/low mitochondrial priming, which are considered as difficult-to-treat^26^. In addition, Raptinal could be unaffected by most mechanisms that mediate primary or acquired resistance to BH3 mimetics. For example, the latter can loose their mitochondria-priming function when binding to BCL-2 proteins is reduced, expression levels of directly BAX-activating proteins (such as BIM) decrease or nontargeted BCL-2 pro-survival proteins are upregulated^27–30^. In stark contrast, Raptinal bypasses BAX/BAK (and also BOK) and is self-sufficient for MOMP induction. Reaching this point of no return irrevocably condemns a cell to death: either via intrinsic apoptosis by caspase-9-dependent activation of downstream effector caspases or loss of mitochondrial function (Fig. 8e). Admittedly, further in vivo studies are needed to estimate the risk for clinically unacceptable side-effects of Raptinal. In combination with novel drug delivery concepts (e.g., conjugation to target-directing antibodies), the tremendous death-inducing potential could perspectively be therapeutically exploitable.

In sum, we show that Raptinal bypasses coordination/initiation of MOMP by pore-forming BCL-2 family proteins^4^. Raptinal exerts a dual cytotoxic effect by rapid activation of the intrinsic apoptotic pathway and simultaneous shutdown of mitochondrial function.

## Material and methods

### Cell lines, antibodies, and reagents

HCT116 cells were obtained from the German Collection of Microorganisms and Cell Culture (DSMZ, Braunschweig, Germany). HCT116 BAX/BAK DKO, BAX KO, and caspase-8 KO cells were kindly provided by Richard Youle (National Institutes of Health, Bethesda, USA), Bert Vogelstein (John Hopkins University, Baltimore, MA, USA) and Hamsa Puthalakath (La Trobe University, Bundoora, Australia), respectively^31–33^. BAX/BAK/BOK-deficient HCT116 cells and MEFs were kindly provided by Thomas Kaufmann (Institute of Pharmacology, University of Bern, Bern, Switzerland). SW48 and DLD1 cells and BAX/BAK-deficient variants thereof were purchased from Sigma (Steinheim, Germany). PCI-1 cells were a gift from Richard Bauer (University of Regensburg, Germany). Jurkat J16 cells and caspase-9- or BAX/BAK-deficient variants thereof have been described before^34^. All cell lines were maintained in RPMI 1640 medium (PAN Biotech, Aidenbach, Germany) with 10% (v/v) fetal calf serum (Sigma). Medium of Jurkat cells was supplemented with 100 U penicillin/mL and 0.1 mg streptomycin/mL (PAN Biotech). Antibodies: caspase-3 (#9662), caspase-9 (#9502), COX IV (#4844), p70S6k (#2708): Cell Signaling (Beverly, MA, USA); tubulin (#MS-581): Dunnlab (Asbach, Germany); cytochrome c (ab13575): abcam (Cambridge, UK). Monoclonal rabbit anti-BOK (BOK-1-5) was a kind gift from Thomas Kaufmann (University of Bern, Bern, Switzerland)^35^. Chemicals: Raptinal and MTT (3-[4,5-dimethylthiazol-2-yl]−2,5-diphenyl tetrazolium bromide): Biomol, (Hamburg, Germany); zVAD-fmk (carbobenzoxy-valyl-alanyl-aspartyl-(Omethyl)-fluoromethylketone): Bachem, (Bubendorf, Switzerland); zLEHD-fmk: BD Biosciences (Heidelberg, Germany); ABT-199, ABT-737, S63845 and QVD-OPh: Hycultec (Beutelsbach, Germany); TRAIL: Apronex (Jesenice u Prahy, Czech Republic); cOmplete protease inhibitor cocktail: Roche (Mannheim, Germany).

### MTT-based cell viability assay

Cells were seeded in 96-well plates (Jurkat: 2 × 10^5^ cells/well; all other cell lines: 2 × 10^4^ cells/well) and challenged with the indicated concentrations of the indicated substances in duplicates (technical replicates). Unless indicated otherwise, cell viability was determined 18 h after stimulation using MTT staining (2 h at 37 °C). Staining intensity was measured at 595 nm and the mean was calculated from the technical replicates of each experiment. The mean value for untreated controls was set to 100%. For any other condition, the MTT staining intensity is given relative to the corresponding untreated group (% of control). Data points shown are mean values (calculated from 2 technical replicates) of independent experiments (*n* ≥ 2–3).

### Western blot analysis

Cells were harvested by centrifugation and lysed in 4× Laemmli sample buffer (8% (w/v) SDS, 0.1 M dithiothreitol, 40% (v/v) glycerol, 0.2 M Tris, pH 8.0) supplemented with phosphatase inhibitor cocktails-I and -II (Sigma). Samples were sonicated and boiled for 5 min at 96 °C before proteins were separated by SDS-PAGE and transferred to PVDF membranes. To block nonspecific binding sites, membranes were incubated in TBS containing 0.1% (v/v) Tween 20 and 5% (w/v) dry milk before primary antibodies of the specificity of interest were added. Antigen-antibody complexes were visualized using horseradish peroxidase-conjugated secondary antibodies (Dako, Hamburg, Germany) and ECL technology (Pierce, Rockford, IL, USA).

### Cytochrome c release by immunoblot

Cytochrome c release by immunoblot was performed essentially as described previously^13^. In brief, 3 × 10^6^ cells were treated with Raptinal for the indicated periods of time. Cells were harvested, centrifuged (1000 × *g*, 2 min), washed with ice-cold PBS, resuspended in 200 μL ice-cold digitonin permeabilization buffer (75 mM NaCl, 1 mM sodium phosphate monobasic, 8 mM sodium phosphate dibasic, 250 mM sucrose, 190 μg/mL digitonin, protease cocktail inhibitor, pH 7.5) and incubated on ice for 5 min. Following centrifugation (14,000 × *g*, 5 min), 150 μL of the supernatant (cytosolic fraction) was collected. The pellet (mitochondrial fraction) was washed in 200 μL digitonin permeabilization buffer and lysed in 25 μL RIPA lysis buffer (150 mM NaCl, 25 mM Tris, 1% (v/v) Nonidet P-40, 1% (w/v) sodium deoxycholate, 0.1% (w/v) SDS, pH 7.5). Forty micrograms of the cytosolic fraction and 50 μg of the mitochondrial fraction were resolved by SDS-PAGE. The cytosolic fraction was probed for tubulin, and the mitochondrial fraction was probed for cytochrome c oxidase subunit IV (COX IV) to confirm equal loading.

### Caspase activity assays

Caspase activity was measured using the caspase-3/-7 activity kit (AAT Bioquest, Sunnyvale, CA, USA) according to manufacturer’s instructions. Emitted fluorescence was quantified using a Victor3 Multilabel Reader (Perkin Elmer, Waltham, MA, USA).

### Flow cytometry

Cell death was assessed by annexin-V and 7-aminoactinomycin D (7-AAD) staining. In brief, HCT116 and Jurkat cells were challenged with 10 µM Raptinal for 15, 30, and 45 min in the presence and absence of 100 µM zVAD-fmk. Afterwards, cells were stained with 7-AAD and annexin-V (4 °C for 15 min in the dark) and analyzed immediately using a FACSCanto flow cytometer (BD Biosciences) following standard procedures^36^. Mitochondrial membrane potential was measured using the MitoScreen Kit (#551302, BD Biosciences) according to manufacturer’s instructions.
